# Safe and Successful Surgical Outcome in Persons with Hemophilia A with and without Inhibitors Treated with Emicizumab: A Large, Single Center, Real-World Experience

**DOI:** 10.3390/jcm12062317

**Published:** 2023-03-16

**Authors:** Giancarlo Castaman, Silvia Linari, Lisa Pieri, Christian Carulli, Paolo Prosperi, Paolo Tonelli, Francesco Demartis, Rajmonda Fjerza, Monica Attanasio, Mirella Coppo, Francesca Salvianti

**Affiliations:** 1 Center for Bleeding Disorders and Coagulation, Department of Oncology, Careggi University Hospital, 50134 Florence, Italy; 2 Department of Orthopedic Surgery, University Hospital of Florence, 50139 Florence, Italy; 3 Emergency Surgery Department, Careggi University Hospital, 50134 Florence, Italy; 4 Department of Surgery and Translational Medicine, University of Florence, 50121 Florence, Italy

**Keywords:** hemophilia A, FVIII inhibitors, FVIII concentrates, recombinant FVIIa, aPCC, prophylaxis

## Abstract

Emicizumab is a humanized recombinant bispecific antibody, bridging together activated factor IX (FIXa) and factor X (FX), thus mimicking the activity of FVIII in vivo. Emicizumab is designed for long-term prophylaxis in patients with severe hemophilia A with and without inhibitors. This approach provides constant protection, with significant reduction in bleeding rate and improved quality of life. However, protection provided by emicizumab is not absolute, and clotting factor concentrates (FVIII, rFVIIa, aPCC) may be necessary for post-traumatic bleeding or surgery, with a potential thrombotic risk or difficulty in preventing bleeding. Real world evidence is still scanty, especially for managing major surgery. In this study, 75 surgeries were managed in 28 patients (27 major procedures in 15 patients and 48 minor procedures in 20 patients. In 17 patients without inhibitors, 30 minor surgeries were carried out by using FVIII in 5, with only a bleeding event, which was successfully treated with FVIII concentrate. Six major surgeries were uneventfully performed with FVIII concentrate. Eleven PWHA and high-titer inhibitors underwent 39 surgical procedures (18 minor and 21 major surgeries). Minor surgeries were mostly performed without prophylaxis with rFVIIa, with only a single bleeding complication. All 21 major surgeries were covered with a homogeneous protocol using rFVIIa. In four instances, bleeding complications occurred, treated with rFVIIa. Of them, a single patient only failed to respond and died because of an uncontrollable bleeding from a large ruptured retroperitoneal pseudotumor. Surgery in patients with emicizumab can be safely carried out with the use of appropriate replacement therapy protocols.

## 1. Introduction

The management of surgical procedures in hemophilic patients has always represented a major concern, being a potential cause of morbidity and mortality. In persons with severe hemophilia A (PWSHA), an intensive treatment with factor VIII (FVIII) concentrate is required together with a postoperative close monitoring of circulating FVIII to maintain protective levels for bleeding, while avoiding excessive levels with inherent thrombotic risk. Even more troublesome are surgeries in PWSHA with inhibitors (PWSHAIn), in which the use of by-passing agents (BPAs), activated prothrombin complex concentrates (aPCC) or recombinant activated FVII (rFVIIa), is necessary, without a suitable laboratory monitoring available [1]. The recent increasing widespread use of the bi-specific monoclonal antibody emicizumab for long-term prophylaxis in PWSHA with and without inhibitors has required a reassessment of antihemorrhagic perioperative treatment with BPAs and FVIII concentrates with their potential risks.

Emicizumab (Hemlibra, Roche Genentech, South San Francisco, CA, USA) is a humanized recombinant bispecific antibody with specificity for activated factor IX (FIXa) and factor X (FX) [2]. It acts as a bridge between FIXa and FX, mimicking the activity of FVIII in vivo and thus enabling generation of activated FX (FXa), which is required for the conversion of prothrombin to thrombin. Emicizumab binding affinity for FIXa and FX is 11-fold lower than that of FVIIIa, thereby reducing the risk of non-localized coagulation; when emicizumab is used, the thrombotic risk may increase as a function of FIXa concentration [3].

The pivotal HAVEN 1 [4] and 2 [5] studies led to emicizumab being granted approval for use in adults and children with severe hemophilia A and inhibitors in November 2017 by FDA and in February 2018 by EMA. These trials reported excellent safety and very low bleeding rates in patients above and below the age of 12 years, respectively. The subsequent HAVEN 3 [6] and 4 [7] studies showed similar results in patients without inhibitors and approval was granted for non-inhibitor patients in October 2018 by the FDA and January 2019 by EMA.

Emicizumab is designed for long-term prophylaxis treatment, providing a constant protection, with significant reduction in annualized bleeding rate (ABR) and improved quality of life (QoL). However, protection provided by emicizumab is not absolute and association with clotting factor concentrates (FVIII, rFVIIa, aPCC) may be necessary in post-traumatic bleeding or surgery, with a potential thrombotic risk. Concomitant use of emicizumab and FVIII concentrates has not resulted in thrombotic events, as FVIII has a greater affinity for IXa and FX, thus displacing emicizumab. According to the Italian Association of Hemophilia Centers (AICE) recommendations on the dosage for the first FVIII infusion as prophylaxis of surgical bleeding in PWSHA on emicizumab, the ranges are the following: 50–100 IU/kg for major surgery, 30–40 IU/kg for minor surgery and 40–50 IU/kg for high-risk endoscopic procedures, respectively [8]. On the other hand, the experience of BPAs and emicizumab co-administration is different. Due to 5 thrombotic episodes, of which 3 thrombotic microangiopathies (MPA) and 2 thromboembolic (TE) events, observed in HAVEN 1 trial [4] in patients receiving aPCC at doses ≥ 100 IU/kg/day for 24 h or longer to treat breakthrough bleeds, the indication to avoid or significant reduction in aPCC dosing, containing FIXa, is commonly accepted. On the contrary, no thrombotic events have been described with the concomitant use of emicizumab and rFVIIa and therefore rFVIIa, at a dose of 100 ± 20 μg/kg, represents the first-line option as replacement therapy in PWSHA with inhibitors on emicizumab [9,10]. Across HAVEN 1–4 trials [4,5,6,7,11], overall, 233 surgeries were performed in 126 out of 399 enrolled patients. Among these, 18 were major and 215 minor surgeries [12]. Most major surgeries (83.3%) were managed with prophylactic coagulation factor and did not result in post-operative bleeds, while 65.6% of minor surgeries/procedures were managed without prophylactic coagulation factor and in 90.8% were not associated with treated bleeds. Early real-world data [13,14,15,16] support clinical trial evidence, showing that surgical procedures can be performed safely in PWSHA treated with emicizumab, regardless of the presence of inhibitors, age, and other risk factors. However, experience in major surgery, in particular orthopedic, is still scanty and worthy to be reported.

Here, we report our experience in management of 75 surgical procedures in PWSHA with and without inhibitors on emicizumab prophylaxis.

### 1.1. Patients and Methods

Data were collected for all patients receiving emicizumab prophylaxis and undergoing surgery between January 2018 and November 2022 at the Center for Bleeding Disorders and Coagulation, Careggi University Hospital, Florence, Italy. All patients gave informed consent, and the study protocol was approved by the local institutional ethics committee.

Demographic data and medical histories were collected from patients’ medical records. Data collection included: age, severity of hemophilia, F8 genotype, FVIII inhibitor status with historical peak and titer at surgery, participation in a multinational clinical trial (HAVEN 1, Stasey or HAVEN 3) of emicizumab. Data on the pre-, intra- and post-operative management and treatment of PWSHA were collected, including type of surgical procedure, planned and unplanned use of hemostatic agents for the peri-operative treatment plan (including product, dose and number of infusions) as well as any treatment plan modifications. For patients with inhibitors undergoing major surgeries, monitoring of prothrombotic markers (Peripheral blood smear for schistocyte detection, platelet count, LDH) were carried out daily.

According to Santagostino et al. [17], surgical procedures were classified as major or minor. Major surgery was defined as an invasive operative procedure where 1 or more of the following occurred: a body cavity was entered, a mesenchymal barrier was crossed, a fascial plane was opened, an organ was removed and/or normal anatomy was operatively altered. Minor surgery was defined as an invasive operative procedure in which only skin, mucous membranes or superficial connective tissue was manipulated.

Information regarding procedure-related bleeding was also collected, including the number of major and clinically relevant non-major bleeds and red blood cell (RBC) transfusion requirements.

Major bleeds were defined according to International Society on Thrombosis and Haemostasis (ISTH) Control of Anticoagulation Subcommittee recommendations [18]. Adverse events and hospitalizations were also recorded.

When clinically appropriate, FVIII activity levels were obtained using a bovine chromogenic assay for measurement of FVIII replacement levels (Chromogenix Coamatic, Instrumentation Laboratory, Bedford, MA, USA on ACL Top550 instrument, Instrumentation Laboratory, Bedford, MA, USA) [19]. In PWSHA with inhibitors a chromogenic Bethesda assay with bovine reagents was used to titer the antibody before surgeries.

Emicizumab concentration was checked prior to major surgeries.

All orthopedic surgeries were performed by a single surgeon.

### 1.2. Data Analyses

All data were retrospectively analyzed and all analyses were regarded as exploratory and descriptive.

## 2. Results

### 2.1. Patient Characteristics and Surgery Overview

Overall, 75 surgeries were managed in 28 patients, with 27 major procedures in 15 patients and 48 minor procedures in 20 patients.

All patients had severe hemophilia A (FVIII activity <1 U/dL), 17/28 patients were without present or past inhibitors to FVIII and the remaining 11 had inhibitors. At the time of surgery inhibitor titer was >5 BU in 5/11 patients and >500 BU in two of them. All patients had previous evidence of significant anamnestic response. Two patients only had undergone ITI protocol 10 years and 5 years before, without success. *F8* gene variants were previously identified in all the patients by using Denaturing High Performance Liquid Chromatography (DHPLC) and direct sequencing [20]. Inversion 22 (IVS-22) analysis was performed by Long PCR, as previously described [20].

Patients’ age ranged from 2 to 65 (median 54) years.

### 2.2. Surgeries in PWSHA and Inhibitors

**Figure 1:**
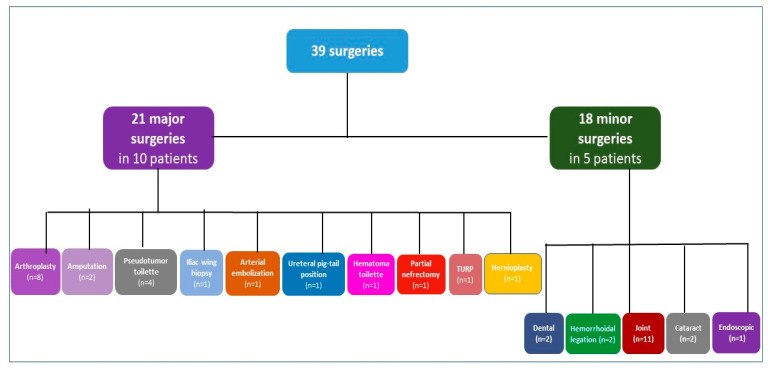
Types of surgeries in patients with hemophilia A and inhibitors. n refers to the number of surgeries. Patients with both minor and major surgeries are counted in both categories; TURP transurethral resection of the prostate.

Eleven PWHA and high titer inhibitors underwent 39 surgical procedures (21 major surgeries, 18 minor surgeries) (Figure 1).

Among the 21 major surgeries in 10 PWSHA with inhibitors, orthopedic procedures are the predominant with 8 arthroplasties, of which 2 total knee replacements (TKR), 1 TKR revision, 3 total hip replacements (THR), 2 THR revisions, further one muscle biopsy of a thigh infected pseudotumor followed by 3 partial excisions of pseudotumor mass and final amputation of the thigh, the other amputation of a thigh was due to advanced unmanageable infected TKR and 1 iliac wing biopsy. One ureteral pig-tail position, one lumbar arterial embolization to treat iliopsoas hematoma and finally one retroperitoneal vast pseudo-tumor iliopsoas hematoma toilet were performed in the same patient undergoing Iliac wing biopsy; one partial nephrectomy for neoplastic lesion, one transurethral resection of the prostate (TURP) and one inguinal hernioplasty were performed in two patients.

The following 18 minor surgeries were performed in 5 patients: 2 tooth extractions, 2 hemorrhoidal ligations, 11 hyaluronic acid joint injections, 2 cataracts and 1 endoscopic removal of bladder stones.

All major orthopedic surgeries were planned to be managed with rFVIIa in addition to emicizumab prophylaxis. All patients were treated with 2–3 bolus of 90 µg/kg rFVIIa every 3 h at the beginning of surgery till wound suturing, followed by FVIIa 90 µg/kg every 4 h during the first 2 days, every 6 h on days 3–4, every 8 hrs on days 5–7, twice a day on days 8–14 and daily (before the rehabilitation, when required) on days 15–20, then rFVIIa was discontinued. Shorter period was adopted for the other surgeries (5–7 days).

Adjunctive intravenous tranexamic acid (TA) 1 g every 12 h for 7 days was administered, except for TURP and inguinal hernioplasty performed in the same surgical session, along with also endoscopic removal of bladder stones. Thromboprophylaxis was not prescribed (Table 1).

Minor surgeries were managed without rFVIIa prophylaxis, except for the endoscopic removal of bladder stones, performed in the same surgical session with two major surgeries, and two tooth extractions. A bolus of rFVIIa (90 µg/kg) was infused before procedure, followed by oral TA (1 g every 8 h for 5 days) (Table 2).

Both in major and minor surgeries, bleeding was as expected depending on the type of procedure and blood transfusions required in 4/19 high-risk major surgeries. Two patients had hip surgery, often followed by transfusion requirement even in normal subjects, one had a leg amputation above the knee and one had severe bleeding already ongoing at the beginning of surgery for a large ruptured retroperitoneal hematoma which ultimately led to the death of the patient. Following one of the two hemorrhoidal varices ligations, the patients had a bleeding, which required a bolus of rFVIIa (90 µg/kg) and TA for 4 days.

No TE events, TMA or significant changes of thrombophilia/microangiopathy markers were observed. Hospitalization days remained consistent with the type of surgery and patient complexity, ranging from 0 to 41 days. The patient, who was admitted for 41 days, died from complications related to recurrent retroperitoneal pseudotumor bleeding.

All minor surgeries did not require hospitalization, except for the endoscopic removal of bladder stones, which was performed in a single operation with TURP and inguinal hernioplasty.

Compared to the historical use of rFVIIa for similar surgeries, a 40% reduction in its use was observed.

### 2.3. Surgeries in PWSHA without Inhibitors

**Figure 2:**
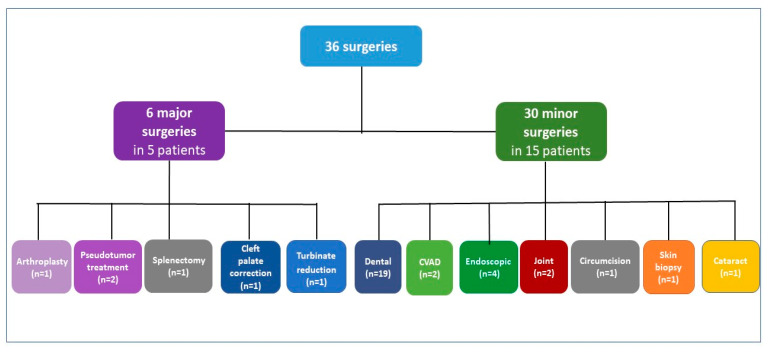
Types of surgeries in patients with severe hemophilia A without inhibitors. n refers to the number of surgeries. Patients with both minor and major surgeries are counted in both categories; CVAD, central venous access device.

Seventeen PWHA without inhibitors underwent 36 surgical procedures (6 major surgeries, 30 minor surgeries) (Figure 2). The following six major surgeries were performed in five of them: one TKR with forced extension on post-operative day 9, one muscle biopsy of a large thigh pseudotumor and subsequent excision of the entire pseudotumor mass, one splenectomy, one cleft palate correction (second operation) and one turbinate reduction.

Among the 30 minor surgeries in 15 PWSHA, dental procedures are the most prevalent (19/30 in 10 patients), including 2 extractions and 4 dental extractions and implants; further 2 portal removals in two children, 4 endoscopic procedures including 2 gastro-duodenal endoscopies with biopsy, 1 colonoscopy and 1 cystoscopy in 2 PWSHA, 2 elbow synoviorthesis with rifampicin in 2, and finally, 1 circumcision for phimosis, 1 skin biopsy and 1 cataract. All major surgeries were managed under FVIII coverage, with factor activity levels of at least 50 IU/dL maintained for 7–14 days. Adjunctive intravenous TA 1 g every 12 h for 7 days was administered. Thromboprophylaxis was not prescribed. Depending on the type of surgery, different treatment schemes with FVIII concentrate have been prescribed, as shown in Table 3.

Minor surgeries were managed with a FVIII preoperative bolus (25–60 IU/kg), except for tooth extractions, dental procedures, and cataract, performed without FVIII coverage. In dental surgeries TA has been used as mouthwash (Table 4).

Both in major and minor surgeries, bleeding was as expected depending on the type of procedure and no transfusional support was needed. Only after one dental extraction a bleeding occurred and required treatment with FVIII (25 UI/ kg × 3 days) in addition to TA. No thromboembolic events occurred. Hospitalization days remained consistent with the type of surgery, ranging from 0 to 15 days. All minor surgeries did not require hospitalization, except for port removals, after which the two children were observed one night, and a gastro-duodenal endoscopy performed for hematemesis from gastritis, when the patient was admitted for 5 days.

In the patient undergoing TKR and post-operative forced extension, the rehabilitation was carried out without FVIII coverage, but only with emicizumab prophylaxis, without bleeding or other complications.

## 3. Discussion

This study represents the largest experience of major orthopedic surgeries in PWHAIn on emicizumab using an internal developed protocol with rFVIIa. According to HAVEN1-4 trials and early real-world data [12,13,14,15,16,21], also in our experience surgical procedures could be safely performed in PWSHA with and without inhibitors treated with emicizumab.

In PWSHA and inhibitors major surgery with a regimen of emicizumab and rFVIIa co-administration has been safely and efficaciously performed. Even two major surgeries and one minor surgery were performed in the same surgical session without complications. The use of bolus doses of rFVIIa was decided to avoid persistent circulating high FVIIa levels with continuous infusion (which is, however, off-label in Italy), since when we planned the protocol the potential thrombotic risk associated with continuous presence of emicizumab and rFVIIa was not completely understood, especially in major orthopedic procedures.

Due to its effectiveness, this protocol was also used in patients with low-titer inhibitor at time of surgery, but with history of high-titer inhibitor. In this setting, FVIII concentrate as peri-surgical coverage could have been administered. However, all these patients had previous history of an anamnestic response and thus we preferred to keep FVIII as a rescue treatment for uncontrolled bleeding, which never occurred in these patients. It should be borne in mind that these patients were recruited over a period of five years and at the beginning knowledge of safety and efficacy of combined treatment of emicizumab and by-passing agent was poorly available, and concerns on the use of aPCC had already been raised. Another important aspect in the homogeneity of the described experience is that all orthopedic surgeries were performed by a single surgeon expert in hemophilia orthopedic surgery.

Also in our experience, minor surgeries were safely performed without prophylaxis with rFVIIa. In particular, the multiple intra-articular administrations of hyaluronic acid were performed without complications. The management of rifampicin intra-articular infiltrations to obtain a chemical synoviorthesis has been instead different, as a covering with clotting factor before the procedure has been carried out in PWSHA without inhibitors.

In PWSHA major surgery with a regimen of emicizumab and rFVIII co-administration has been safely and efficaciously performed, as minor surgeries also without prophylaxis with rFVIII.

Our data enlarge real-world real-world experience and the results from previous clinical trials. However, these studies reported a large prevalence of minor surgeries and few major surgeries, especially orthopedic. In a retrospective analysis of PwHA with/without FVIII inhibitors on emicizumab, 31 surgeries (29 minor, 2 major) were performed in 25 patients [21]. As in our experience, minor surgeries were managed with emicizumab alone or with additional FVIII or rFVIIa and tranexamic acid and major bleeding occurred in one instance only in a patient treated with tranexamic acid alone. Post-operative FVIII was used for hip replacement in a patient without inhibitor, while an explorative laparotomy, was managed with additional rFVIIa in a patient with inhibitor. An additional real-world experience reported 20 minor and 5 major surgeries in 22 PwHA with/without FVIII inhibitors [14]. All major surgeries were performed with additional hemostatic agents, while only a minority of minor surgeries (≈20%) required additional treatment. No relevant bleeding episodes were reported, and no thrombotic complications occurred. In an observational study, 28 minor surgeries and 2 major surgeries were reported in 22 PwHA with/without FVIII inhibitors with similar results and no thrombotic events [13]. Most of surgeries were represented by 21 CVAD removals. Even in the large experience reported with HAVEN 1–4 studies [12], the number of patients undergoing arthroplasty was lower than in the present study. In those studies, however, inclusion criteria were stringent and planned surgery represented an exclusion criteria. In our experience major orthopedic surgery in patients with inhibitors was safely carried out, with very few bleeding complications easily managed by using rFVIIa. No significant complications occurred in patients without inhibitors.

The experience accumulating on surgery in patients with emicizumab spans at least 5 years and the present evidence is reassuring about the safe and effective use of replacement treatment in these patients, even with rFVIIa, and the excellent outcome. This experience supports a more confident approach to these clinical situations, in the past considered critical especially for patients with inhibitors. However, a multidisciplinary team and strict clinical and laboratory monitoring are always recommended for the management of surgical procedures in PWSHA with and without inhibitors treated with emicizumab.

## Figures and Tables

**Table 1:** Major surgeries in patients with inhibitors.

Patient N.	Surgery N.	Age (yrs)	Pathogenic Mutation	Historical FVIII INH Peak (BU/mL)	FVIII INH at Surgery (BU/mL)	Surgery Type	rFVIIa Use °	TA Use *	Bleeding Episode	Transfusion Requirement	Days of Hospitalization
1	1	59	EX 23-26 deletion	70	2	THR	Yes	Yes	Yes	Yes (2 RBC)	12
1	2	62	-	70	1.8	TURP	Yes	No	No	No	5
1	3	62	-	70	1.8	Inguinal hernioplasty	Yes	No	No	No	5
2	4	56	EX 15 c.1727 del3bp ins22bp	7.9	2.8	Amputation of a thigh	Yes	Yes	No	No	12
2	5	57		7.9	4.4	TKR	Yes	Yes	No	No	15
3	6	49	EX 2-25 deletion	840	760	TKR revision	Yes	Yes	No	No	12
3	7	49	-	840	680	Revision THR	Yes	Yes	Yes	Yes (4 RBC)	23
4	8	61	IVS-22	UK	2.9	Revision THR	Yes	Yes	No	No	12
5	9	62	IVS-22	UK	6.1	Pseudotumor of thigh biopsy	Yes	Yes	No	No	4
5	10, 11, 12	62	-	UK	6.1	Pseudotumor of thigh removal	Yes	Yes	No	No	15
5	13	62	-	UK	6.1	Amputation of a thigh	Yes	Yes	Yes	3	32
6	14	58	EX 7-13 deletion	16,400	2.5	Iliac wing biopsy	Yes	Yes	No	No	41
6	15	58	-	16,400	2.5	Lumbar arterial embolization	Yes	Yes	No	No	41
6	16	58	-	16,400	2.5	Ureteral pig-tail positioning	Yes	Yes	No	No	41
6	17	58	-	16,400	2.5	Retroperitoneal hematoma curettage	Yes	Yes, for 14 days	Yes	Yes (5 RBC, 10 FFP, 2 PC)	41
7	18	64	p. Arg427X	154	15	Partial nefrectomy for renal cancer	Yes	Yes	No	No	7
8	19	42	EX 5-13 deletion	1200	15	TKR	Yes	Yes	No	No	12
9	20	49	IVS 22	32	0.5	THR	Yes	Yes	No	No	12
10	21	56	p.Leu264Gln	41	0.5	THR	Yes	Yes	No	No	15

Legenda: INH, inhibitor; THR, total hip replacement; TKR, total knee replacement; TURP, transurethral resection of the prostate; rFVIIa, recombinant activated factor VII; ° (2–3 bolus of 90 µg/kg rFVIIa every 3 h at the beginning of surgery till wound suturing, followed by FVIIa 90 µg/kg every 4 h during the first 2 days, every 6 h on days 3–4, every 8 h on days 5–7, twice a day on days 8–14 and daily, in case of rehabilitation, on days 15–20); TA, Tranexamic acid; * (1 g every 12 h for 7 days); RBC, red blood concentrate; FFP, fresh frozen plasma; PC, platelet concentrate.

**Table 2:** Minor surgeries in PWSHA and inhibitors.

Patient N.	Surgery N.	Age (yrs)	Pathogenic Mutation	FVIII INH Historical Peak (BU/mL)	FVIII INH at Surgery (BU/mL)	Surgery Type	Pre-Operative Factor Treatment	Post-Operative Factor Treatment	TA Use	Bleeding Episode	Hemostatic Treatment	Days of Hospitalization
1	1, 2	59	EX 23-26 deletion	70	2	Intra-articular administration of hyaluronic acid (N.2)	No	No	No	No	No	0
1	3, 4	60		70	2	Cataract (N.2)	No	No	No	No	No	0
1	5 *	62		70	1.8	Endoscopic removal of bladder stones *	Yes *	Yes *	No	No	No	5 *
2	6, 7, 8, 9, 10, 11, 12, 13, 14	42	EX 5-13 deletion	1200	15	Intra-articular administration of hyaluronic acid (N.9)	No	No	No	No	No	0
3	15, 16	56	EX 15 cod 1727 del3bp ins22bp	7.9	4.4	Hemorrhoidal varices ligation (N.2)	No	No	No	Yes, in surgery N.15	FVIIa 90 µg/kg + TA 1 g every 8 h for 4 days	0
3	17	56	EX 15 cod 1727 del3bp ins22bp	7.9	4.4	Dental extraction	FVIIa 90 µg/kg	No	Mouth wash with TA 0.5 g every 8 h for 7 days	No	No	0
4	18	32	EX 14-27 deletion	10,200	520	Dental extraction	FVIIa 90 µg/kg	No	Mouth wash with TA 0.5 g every 8 h for 7 days	No	No	0

Legenda: INH, inhibitor; * Surgery N.5 performed in a single operation with major surgeries N. 2 and N.3; rFVIIa, recombinant activated factor VII.

**Table 3:** Major surgeries in PWSHA without inhibitors.

Patient N.	Surgery N.	Age (yrs)	Pathogenic Mutation	Surgery Type	Pre-Operative Factor Treatment	Post-Operative Factor Treatment	Adjunctive Antifibrinolytics	Bleeding Episode	Transfusion Requirement	Days of Hospitalization
1	1	28	p.Trp1854Cys	TKR with forced extension on post-operative 9 day	SHL-rFVIII 70 IU/kg	43 IU/kg every 12 h × 3 days; 43 IU/kg every 24 h × 5 days; 43 IU/kg every 12 h × 1 day; 28 IU/kg every 12 h × 3 days; 14 IU/kg every 12 h × 2 days; 14 IU/kg every 24 h × 7 days	TA 1 g i.v. every 12 h × 7 days	No	No	15
2	2	63	Arg1689Cys + Asp1241Glu	Pseudotumor of thigh biopsy	SHL-rFVIII 66 IU/kg	50 U/kg every 24 h × 3 days; 50 U/kg every 48 h × 4 days	TA 1 g i.v. every 12 h × 7 days	No	No	0
2	3	63	Arg1689Cys + Asp1241Glu	Pseudotumor of thigh excision	SHL-rFVIII 85 IU/kg	50 IU/kg every 12 h × 1 day; 30 IU/kg every 12 h × 6 days; 50 IU/kg every 24 h × 7 days	TA 1 g i.v. every 12 h × 7 days	No	No	7
3	4	47	IVS-1	Splenectomy	SHL-rFVIII 70 IU/kg	35 IU/kg every 12 h × 2 days; 23 IU/kg every 12 h × 4 days; 35 IU/kg every 24 h × 7 days	TA 1 g i.v. every 8 h × 7 days	No	No	11
4	5	5	c.[3430del], p.Ser1144ValfsX5	Cleft palate correction (2nd operation)	SHL-rFVIII 55 IU/kg	55 IU/kg every 12 h × 1 day; 55 IU/kg every 24 h × 6 days. 55 IU/kg every 48 h × 7 days	TA 0.5 g orally every 8 h × 14 days	No	No	7
5	6	39	p.Arg2323Cys	Turbinate reduction	EHL-rFVIII 25IU/kg	25 IU/kg every 24 h × 2 days	TA 1 g orally every 8 h × 7 days	No	No	2

Legenda: TKR, total knee replacement; SHL-rFVIII, standard half-life recombinant factor VIII; EHL-rFVIII, extended half-life recombinant factor VIII; IU: International Unit; TA: tranexamic acid; i.v.: intravenous.

**Table 4:** Minor surgeries in patients without inhibitors.

Patient N.	Surgery N.	Age (yrs)	Pathogenic Mutation	Surgery Type	Pre-Operative Factor Treatment	Post-Operative Factor Treatment	TA Use	Bleeding Episode	Hemostatic Treatment	Days of Hospitalization
1	1	47	IVS-1	Intra-articular administration of rifampicin	SHL-rFVIII 25 IU/kg	No	No	No	No	0
1	2	47	-	EGDS for hematemesis	SHL-rFVIII 60 IU/kg	No	1 g orally every 8 h for 7 days	No	No	5
1	3	47	-	Dental extraction	No	No	0.5 g every 8 h for 7 days (mouth wash)	No	No	0
2	4	54	p.Gly469Arg	Intra-articular administration of rifampicin	SHL-rFVIII 25 IU/kg	No	No	No	No	0
2	5	54	-	Dental extraction	No	No	0.5 g every 8 h for 7 days (mouth wash)	Yes	SHL-rFVIII 25 IU/kg × 3 days	0
3	6, 7, 8	63	IVS22	Dental extraction and implant (N.3)	SHL-rFVIII 50 IU/kg	No	0.5 g every 8 h for 7 days (mouth wash)	No	No	0
4	9	62	EX14 ins 1 bp +A 1588-1590	Dental extraction and implant	SHL-rFVIII 50 IU/kg	No	0.5 g every 8 h for 7 days (mouth wash)	No	No	0
5	10, 11	63	p.Arg1689Cys + p.Asp1241Glu	Dental procedure (N.2)	No	No	No	No	No	0
6	12, 13	56	p.Leu308Arg	Dental procedure (N.2)	No	No	No	No	No	0
7	14, 15	18	IVS22	Dental procedure (N.2)	No	No	No	No	No	0
8	16, 17, 18	42	IVS22	Dental procedure (N.3)	No	No	No	No	No	0
9	19, 20	60	C.4379del p.Asn1460Ilefs*5	Dental procedure (N.2)	No	No	No	No	No	0
10	21, 22	57	IVS22	Dental procedure (N.2)	No	No	No	No	No	0
11	23	52	IVS22	Colonscopy	SHL-rFVIII 30 IU/kg	No	No	No	No	0
11	24	52	-	EGDS with biopsy	SHL-rFVIII 45 IU/kg	No	1 g orally every 12 h for 5 days	No	No	0
11	25	52	-	Cystoscopy	SHL-rFVIII 45 IU/kg	No	No	No	No	0
12	26	39	p.Arg2323Cys	Circumcision for phimosis	EHL-rFVIII 25 IU/kg	25 IU/kg every 24 h × 1 day	1 g orally every 8 h × 5 days	No	No	0
13	27	65	p.Asp222_Tyr2351delins13	Cataract	No	No	No	No	No	0
13	28	65	-	Skin biopsy	SHL-rFVIII 50 IU/kg	No	No	No	No	0
14	29	2	p.Arg2182His	Port removal	SHL-rFVIII 50 IU/kg	No	No	No	No	1
15	30	2	IVS22	Port removal	SHL-rFVIII 50 IU/kg	No	No	No	No	1

Legenda: TA, tranexamic acid; SHL-rFVIII, standard half-life recombinant factor VIII; EHL-rFVIII, extended half-life recombinant factor VIII; IU, International Unit; EGDS: esophagogastroduodenoscopy.

## Data Availability

Not applicable.
